# Cesarean scar ectopic pregnancy: A cause of failed first trimester surgical termination of pregnancy (case report)

**DOI:** 10.5935/1518-0557.20200039

**Published:** 2021

**Authors:** Haitham A. Torky

**Affiliations:** 1 Department of Obstetrics & Gynecology - October 6th University & As-Salam International Hospital, Cairo, Egypt

**Keywords:** cesarean scar pregnancy, diagnosis, surgical management

## Abstract

Cesarean scar (ectopic) pregnancy is due to blastocyst implantation on a Caesarean scar. The current case presented by vaginal bleeding after a failed surgical termination of pregnancy. The ultrasound scan revealed a cesarean scar ectopic pregnancy managed by surgical removal. The possibility of cesarean scar ectopic pregnancy should be considered in any case presenting with a low-lying gestational sac.

## INTRODUCTION

Cesarean scar (ectopic) pregnancy is due to blastocyst implantation on a Caesarean scar. It is the least common type of ectopic pregnancies (Herman *et al*., 1995). I am presenting a case of cesarean scar ectopic pregnancy with vaginal bleeding after failed surgical termination of a first trimester pregnancy.

## CASE REPORT

**Figure 1:**
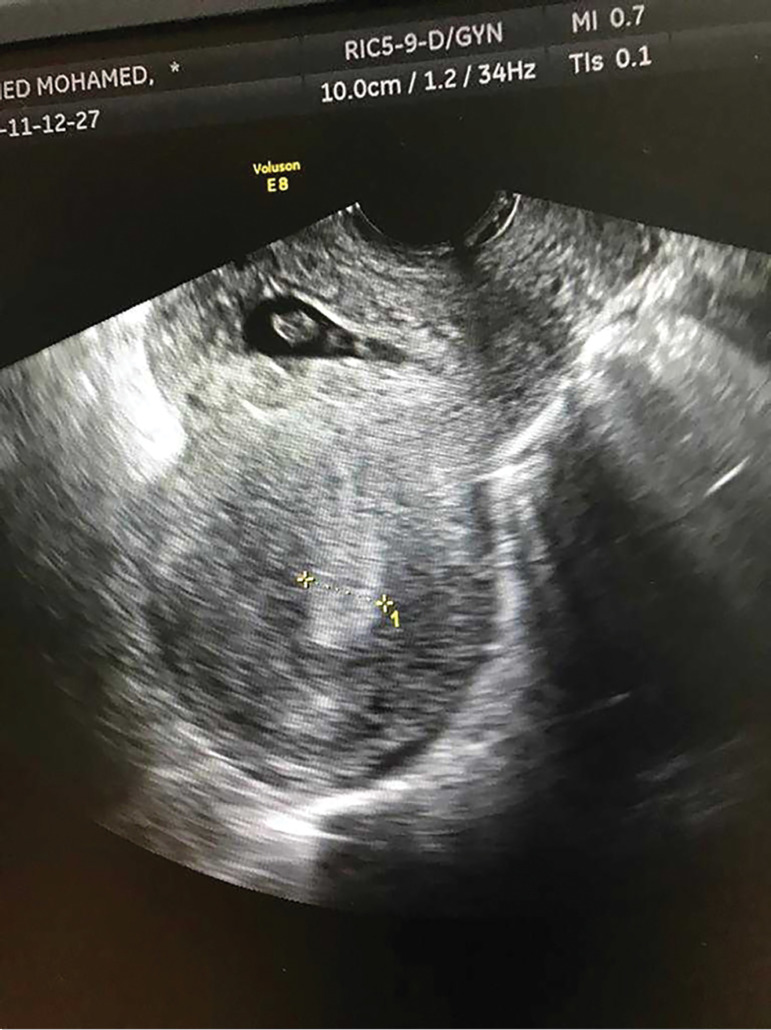
Ultrasound picture showing ballooning of cesarean scar by a pregnancy sac

**Figure 2:**
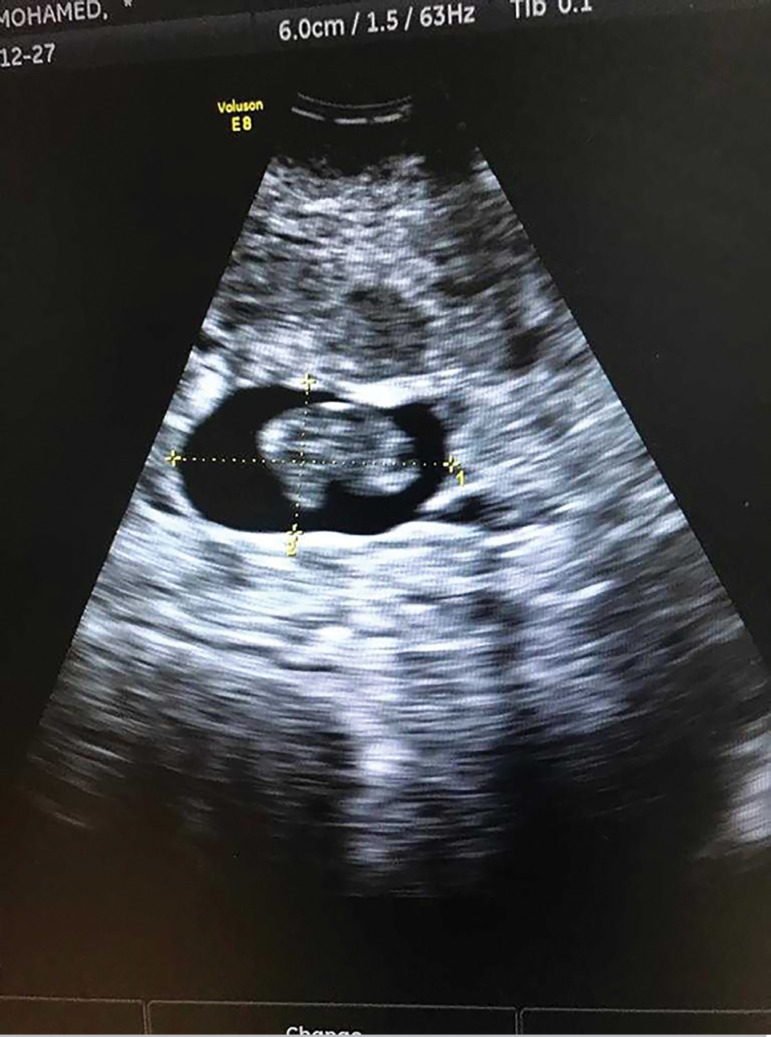
Ultrasound picture showing pregnancy sac with fetal pole

A thirty-nine year old woman Gravida 6 Para 3 - all by cesarean sections - who was 7 weeks pregnant presented to the emergency department with moderate vaginal bleeding following a failed attempt of surgical termination of pregnancy outside the hospital, as she was initially miss-diagnosed as a missed miscarriage. On examination, her pulse was 110, blood pressure 100/60 and temperature of 36.6. Her BhCG was 56,000 mIU/mL and hemoglobin of 12.4 g/dl, while her transvaginal ultrasound revealed an empty uterine cavity and an intact gestational sac with fetal pole but no fetal heart, implanted at the site of the scar causing ballooning of this site (Figures 1 and 2). She was diagnosed as scar ectopic pregnancy. Considering her condition, we decided to do a laparotomy, we cross-matched her blood and obtained a signed informed consent form.

### Operative technique

**Figure 3:**
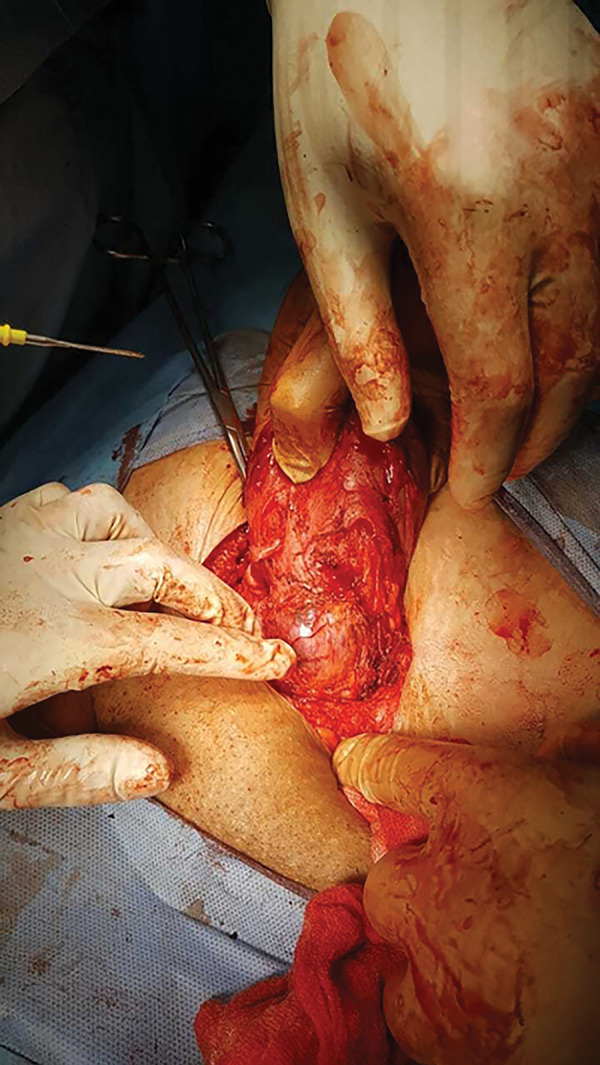
Operative picture of the ballooned scar ectopic pregnancy

We did the laparotomy using the Pfannenstiel skin incision. Her urinary bladder was adherent to the anterior surface of the uterus, near the uterine fundus. We sharply dissected the adhesions until reaching the ballooned scar ectopic site (Figure 3). The uterus was thoroughly inspected for possible perforation by the failed attempt of surgical termination of pregnancy, and then we proceeded with a segmental resection of the area containing the pregnancy followed by suturing the edges to close the defect. We left an intraperitoneal drain, and removed it two days later. Post-operative hemoglobin was 9 g/dl. She made an uneventful recovery and was discharged on hematinic.

## DISCUSSION

Cesarean scar ectopic pregnancy has an estimated incidence between 1/1,800 and 1/2,216 pregnancies (Seow *et al*., 2004). It is a serious condition, which may cause excessive bleeding and uterine rupture.

The rising rate of caesarean sections has increased the risk of some serious conditions as placenta previa, placental abruption, placenta accreta, percreta, and cesarean scar ectopic pregnancies. Theories explaining this condition are blastocyst invasion of the myometrium via a microscopic dehiscent tract resulting from previous uterine surgery, like surgical termination of pregnancies and Cesarean sections (Cignini *et al*., 2007), and trauma caused by assisted reproductive techniques in cases with no previous surgeries (Aich *et al*., 2015).

The commonest presenting symptom of Caesarean scar ectopic pregnancy is painless vaginal bleeding in the absence of clinical signs; however, in the current case, there was bleeding following a failed attempt of surgical evacuation. Transvaginal ultrasonography and color flow Doppler are helpful diagnostic tools (Fylstra *et al*., 2002; Jurkovic *et al*., 2003), because they can differentiate between cesarean scar ectopic and low-lying intra-uterine gestational sac, thus avoiding a faulty intervention which resulted in vaginal bleeding and could have resulted in uterine perforation and additional morbidity in the current case, during the failed attempt of surgical termination of pregnancy. Cesarean scar ectopic pregnancy should also be differentiated from cervical pregnancies, which are characterized by absence of myometrium between the gestational sac and bladder, because the gestational sac grows into the anterior wall of the isthmus (Fylstra, 2012). To determine whether a Cesarean Scar Pregnancy (CSP) has occurred, one can use an ultrasound scan in the sagittal view to indicate a clear uterine cavity and an empty cervical canal (Rizk *et al*., 2013), as shown in Figure 1.

Rizk *et al*. (2013) studied the use of intramuscular and intra-gestational methotrexate in twenty-six cases of suspected ectopic pregnancies - nineteen of them were Caesarean scar ectopic - with successful outcome. After treatment, there was an initial rise in the human chorionic gonadotropin serum level, the volume of the gestational sac and it’s vascularization, followed by a fall in the level of serum human chorionic gonadotropin after a variable period of time; however, this treatment option was not used in the current case, considering the amount of bleeding and high human chorionic gonadotropin level. 

Surgical options were used in several case reports even in the absence of bleeding (Aich e*t al*., 2015). One of those options is laparotomy and excision of the gestational mass as the one used in the current case, this option decreases the risk of recurrence and a shorter follow-up period as the old scar is resected with a new uterine closure (Jurkovic *et al*., 2003; Fylstra, 2012; Maymon *et al*., 2004).

## CONCLUSION

Cesarean scar ectopic pregnancy should be excluded in any case with a low-lying intra-uterine pregnancy sac before performing surgical termination of a first trimester pregnancy as this avoids faulty interventions, which can increase both morbidity and mortality.
